# Measuring phonetic convergence in speech production

**DOI:** 10.3389/fpsyg.2013.00559

**Published:** 2013-08-27

**Authors:** Jennifer S. Pardo

**Affiliations:** Department of Psychology, Montclair State UniversityMontclair, NJ, USA

**Keywords:** phonetic convergence, speech production, speech perception, imitation, conversation

## Abstract

Phonetic convergence is defined as an increase in the similarity of acoustic-phonetic form between talkers. Previous research has demonstrated phonetic convergence both when a talker listens passively to speech and while talkers engage in social interaction. Much of this research has focused on a diverse array of acoustic-phonetic attributes, with fewer studies incorporating perceptual measures of phonetic convergence. The current paper reviews research on phonetic convergence in both non-interactive and conversational settings, and attempts to consolidate the diverse array of findings by proposing a paradigm that models perceptual and acoustic measures together. By modeling acoustic measures as predictors of perceived phonetic convergence, this paradigm has the potential to reconcile some of the diverse and inconsistent findings currently reported in the literature.

The acoustic-phonetic form of an utterance is highly variable due to the influence of multiple factors in speech production. Some of these factors relate to relatively stable aspects of talker physiology, but other factors also play a role in shaping an utterance. These factors include talker dialect and idiolect, as well as relatively transient aspects of a conversational setting. Recent research on phonetic convergence has demonstrated that talkers converge to an interlocutor or to an auditory model on multiple acoustic-phonetic dimensions. Across numerous studies, investigators have assessed convergence in many acoustic attributes, with differing degrees of success and consistency. The current paper reviews research on phonetic convergence in both non-interactive and conversational settings, and attempts to consolidate the diverse array of findings by proposing a paradigm that models perceptual and acoustic measures together. Ultimately, a more comprehensive model of speech communication must integrate social and cognitive approaches to speech production and perception.

## Foundations in social & psycholinguistic approaches

Early research on Communication Accommodation approached speech variability with respect to interpersonal attributes of social settings, yielding a large body of research in this domain (e.g., Giles, 1973; Bourhis and Giles, 1977; Street, 1982; Coupland, 1984; Putman and Street, 1984; Giles et al., 1991; Shepard et al., 2001). In this approach, convergence on a variety of so-called non-content speech attributes was examined in relation to listener evaluations of talker competence and social attractiveness. In the earliest studies, Giles first established both convergence (Giles, 1973) and divergence (Bourhis and Giles, 1977) in degree of regional accentedness in the speech of ordinary Englishmen. This sort of code-switching behavior was attributed to attempts to decrease (or increase) social distance between interlocutors.

As the field matured, other studies examined more detailed acoustic-phonetic attributes. For example, Putman and Street (1984) assessed convergence among same-sex interviewers and interviewees in speaking rate, turn durations, and response rates (inter-turn-intervals during the conversations). The only measure that yielded a significant correlation between talkers was speaking rate (*r* = 0.66), in a condition in which the interviewee was attempting to create an impression of competence and likeability. Coupland (1984) provided an in-depth examination of the use of four phonological variables (h-dropping, t-flapping, ng-dropping, and simplification of final consonant clusters) in conversations between a travel agent and 51 of her clients in Cardiff, England. The frequency of usage of standard vs. non-standard phonological variables for these variables was highly correlated, with coefficients ranging from 0.76 to 0.90. Other attributes yielding significant findings in this research domain included intensity (Natale, 1975) and sub-vocal frequency variation (Gregory and Webster, 1996; Gregory et al., 1997, 2000). In general, this approach related convergence to factors such as social status or likeability, with little consideration of the cognitive mechanisms supporting convergence or divergence.

In the 1990s, psycholinguists began to model talker variability as an integral part of speech perception and memory for words (Mullennix et al., 1989; Mullennix and Pisoni, 1990; Goldinger et al., 1991; Palmeri et al., 1993; Nygaard et al., 1995; Goldinger, 1996; Nygaard and Pisoni, 1998; Bradlow et al., 1999). This endeavor culminated in a seminal report of spontaneous phonetic imitation during speech shadowing (Goldinger, 1998), which inspired a resurgence of interest in phonetic convergence. Along with an intriguing report of long-term “gestural drift” in a single talker (Sancier and Fowler, 1997), it appeared that talkers vary acoustic-phonetic repertoire to sound more similar to a model talker or to a particular linguistic environment. Thus, not only was talker identity an integral part of speech perception, but perception of talker characteristics also influenced speech production. This approach introduced a new paradigm for exploring convergence that avoids some of the pitfalls of employing acoustic-phonetic measures alone.

In Goldinger's (1998) study, talkers provided baseline and shadowed versions of mono- and bi-syllabic words. In order to assess imitation, the baseline and shadowed utterances were used along with the model utterances in an AXB perceptual similarity test. In the AXB task, a separate set of listeners decided which of the two flanking items (A or B), both produced by one talker, sounded like a better imitation of the middle item (X), produced by the model talker. The two items being compared (A & B) comprised baseline and repeated utterances. Imitation was quantified as the percentage of shadowed items that sounded more similar to a model talker's items than baseline items. This study was the first to establish phonetic imitation in speech shadowing, and the patterns of influence of word frequency and repetition on imitation supported an episodic model of word recognition. Once imitation had been established for shadowed speech, two other studies replicated and extended the paradigm to include the influence of talker sex (Namy et al., 2002) and variation in voice onset timing (Shockley et al., 2004) on phonetic convergence in shadowed speech.

Pardo (2006) integrated the earlier social approach with this psycholinguistic paradigm by adapting the AXB perceptual similarity task for use with conversational speech. In contrast to earlier studies on Communication Accommodation, the conversational task did not involve a confederate interviewer or rely on acoustic-phonetic measures. Instead, unacquainted talkers completed a modified version of the HCRC Map Task (Anderson et al., 1991), which comprises pairs of iconic maps with labeled landmarks that talkers use to complete a map navigation task. During the Map Task conversations, the talkers naturally repeated the landmark label phrases—typically, one talker would introduce a landmark and their partner would repeat the landmark label phrase soon after. These repeated task utterances were sampled, along with pre-task (baseline) and post-task (persistence) recordings of the landmark labels, to compose AXB similarity tests for separate listeners. On each trial, a listener heard three versions of a landmark label phrase and decided which of the two flanking items (baseline vs. task repetition) sounded more like the middle item (partner's prior utterance) in pronunciation. Overall, talkers converged during conversation, convergence increased over the course of the interaction and persisted into the post-task session, and talker role and pair sex influenced degree of convergence. Other studies have extended findings of phonetic convergence during conversational speech to examine the influence of a directed imitation task (Pardo et al., 2010), a role-switching task (Pardo et al., 2013a), and dialect similarity (Kim et al., 2011).

An important contribution of this psycholinguistic approach is the use of the AXB perceptual task for measuring phonetic convergence. Perceptual measures provide a more global appraisal of convergence that is not limited to the particular acoustic-phonetic attribute a researcher might choose. First, it is not possible to characterize the phenomenon in a comprehensive manner without including multiple dimensions. Talkers can converge on one dimension at the same time that they diverge or produce random variation in other dimensions. Moreover, this flexibility extends across different items. For example, convergence in F0 on one item does not imply that the talker will always or only converge on F0. On another item, the talker might converge on vowel formants or duration instead. Indeed, this is the kind of pattern that has been found in studies that have examined multiple acoustic attributes (Putman and Street, 1984; Pardo, 2010; Lelong and Bailly, 2011; Levitan and Hirschberg, 2011; Pardo et al., 2012, 2013a,b). When listeners perform an AXB perceptual similarity task, they are effectively collapsing across these multi-dimensional acoustic patterns, providing a more reliable measure of phonetic convergence than any single acoustic-phonetic attribute.

A second reason to include perceptual measures relates to the potential social function of phonetic convergence. According to Communication Accommodation Theory, talkers converge or diverge in order to manage social distance, or to display affiliation (Giles et al., 1991; Shepard et al., 2001). Although a particular acoustic measure might show reliable convergence, it is not necessarily the case that listeners would detect convergence in that attribute. There might be other attributes that diverged, and that are more salient to a listener, or the attribute of interest might be a correlate of another attribute that was more salient to both the listener and the talker who produced the item. Therefore, perceptual assessment of phonetic convergence provides a more valid measure for generalizing to social settings.

There are two main issues to consider when employing perceptual measures. First, just as talkers might converge on different attributes across different items, listeners are not likely to be uniform in their appraisal of similarity. Therefore, it is necessary to collect data from multiple listeners per talker pair, and to incorporate all levels of variability in the analyses. To date, studies have employed from 5 to 30 listeners per pair in perceptual similarity tasks (Goldinger, 1998; Shockley et al., 2004; Pardo, 2006; Pardo et al., 2010, 2012, 2013a,b; Kim et al., 2011; Babel and Bulatov, 2012). The exact number of listeners needed depends on a number of factors, including the power of statistical analyses to be used and the need to generalize across other listeners. Second, the exclusive use of perceptual measures does not provide information about which acoustic-phonetic attributes were employed by the talkers. A more comprehensive approach should include both acoustic and perceptual measures, in which the acoustic measures are used to model the perceptual data in mixed-effects regression analysis (Baayen, 2008; Baayen et al., 2008; Jaeger, 2008; Barr et al., 2013).

## Diverse findings in recent research

Across studies of phonetic convergence that have focused exclusively on acoustic measures, the landscape of potential attributes is very large. Recently, studies employing acoustic-phonetic measures have examined duration (Goldinger, 1998; Mitterer and Ernestus, 2008; Pardo, 2010), speaking rate (Pardo et al., 2010, 2013a) F0 or intonation contour (Gregory et al., 1997; Goldinger, 1998; Pardo, 2010; Lelong and Bailly, 2011; Levitan and Hirschberg, 2011; Babel and Bulatov, 2012), intensity (Levitan and Hirschberg, 2011), voice quality (Levitan and Hirschberg, 2011), vowel spectra (Vallabha and Tuller, 2004; Pardo et al., 2010, 2012, 2013a,b; Lelong and Bailly, 2011; Babel, 2012), voice onset time (Sancier and Fowler, 1997; Fowler et al., 2003; Shockley et al., 2004; Sanchez et al., 2010; Nielsen, 2011), lip aperture (Gentilucci and Bernardis, 2007) and individual phonemic variants (Coupland, 1984; Delvaux and Soquet, 2007; Mitterer and Ernestus, 2008; Honorof et al., 2011). Most studies have only focused on a single attribute, while a few have examined more than one attribute at a time (Goldinger, 1998; Pardo et al., 2010, 2012, 2013a,b; Lelong and Bailly, 2011; Levitan and Hirschberg, 2011).

Although this approach has yielded some positive findings, there is currently no compelling rationale or standard for choosing one acoustic attribute over another. Unfortunately, a reliance on acoustic measures yields datasets that are relatively inconsistent and chaotic. For example, a few studies have examined convergence in vowel formants. In one study, multiple male and female talkers shadowed words produced by 2 male talkers (Babel, 2012). The items varied in target vowel (5 vowels: /i/, /æ/, /a/, /o/, and /u/), allowing for comparison of convergence across the different vowels. Overall, all vowels were found to converge, but /æ/ and /a/ showed the greatest levels convergence. However, this pattern of vowel convergence differs from that reported in two studies of convergence during conversational interaction (Pardo, 2010; Pardo et al., 2010) and in a study of convergence in college roommates (Pardo et al., 2012).

In Pardo (2010), talkers converged on /i/ and /u/ and diverged on /æ/ and /a/, which is nearly opposite the pattern reported by Babel (2012), and neither pattern was found in Pardo et al. (2010). In a study of convergence in college roommates across the academic year, there were no consistent patterns of convergence in vowel formants across pairs of talkers (Pardo et al., 2012). Instead, some talkers converged in average vowel formants over the course of the academic year while others diverged. At the same time, however, perceptual measures of convergence indicated modest phonetic convergence overall that was unrelated to convergence in vowel formants. Likewise, Babel and Bulatov (2012) reported that filtering F0 information reduced degree of shadowing convergence in both F0 and perceptual measures, but that the acoustic F0 measure and the perceptual measure were not correlated with each other. Finally, Vallabha and Tuller (2004) reported that talkers were unable to imitate their own vowels faithfully, exhibiting biased productions that were most likely related to dialectal differences among the talkers.

Investigations of temporal measures such as speaking rate likewise have yielded inconsistent findings. In two studies, Pardo and colleagues examined articulation rate convergence in concert with AXB measures of phonetic convergence (Pardo et al., 2010, 2013a). In both studies, listeners detected convergence when talkers failed to converge in speaking rate. Not only were the between-talker cross-correlation measures of speaking rate near zero, but talkers differed significantly in speaking rates according to their role in the conversation. These findings contrast with early reports of convergence in speaking rate, and they could be due to the differences in the conversational tasks. In early reports by Street (1982) and Putman and Street (1984), the talkers participated in an interview task, whereas Pardo and colleagues used the Map Task, which has a clearly defined role difference between talkers. Thus, neither measures of vowel formants nor speaking rate can be considered diagnostic of phonetic convergence.

In a comprehensive study of convergence on multiple acoustic parameters during conversational interaction, Levitan and Hirschberg (2011) assessed conversational convergence in intensity, pitch, speaking rate, jitter, shimmer, and noise-to-harmonic ratio. They reported four kinds of convergence in all measures: session level proximity of conversational partners, changes in proximity from the first to the second half of the first game in the interaction and for the entire multi-game session, and turn-by-turn synchrony. Overall, intensity was strongest in both proximity and convergence. This finding is not surprising given the well-known Lombard sign (Lane and Tranel, 1971). At the session level, the talkers were more similar to their partners than to others in intensity (mean and max), pitch (max only) and speaking rate. During the first game, talkers converged on intensity (mean only), shimmer, and noise-to-harmonics ratio. Pitch mean and jitter converged over the halves of the entire session, but were not significant in proximity in the second half. Inter-talker correlations in turn-by-turn synchrony, proximity, and convergence for all acoustic measures were significant, but moderate to weak, at 0.50 or less.

This thorough approach yields interesting but chaotic data, and it is not known which acoustic attributes are perceptible to listeners, and which play a relatively minor role. On the one hand, a unit change in intensity is not perceived in the same manor as a unit change in F0/pitch. On the other hand, convergence in one acoustic attribute might offset divergence in another. Perceptual assessment of phonetic convergence provides a common metric for evaluating the relative contribution of individual acoustic attributes. Moreover, any acoustic change that is not perceptible is unlikely to play a social role in interaction (Giles et al., 1991; Shepard et al., 2001).

A final reason to employ more comprehensive measures of phonetic convergence relates to the file-drawer problem in scientific research. Given the nature of acoustic-phonetic variability in general, and across the studies summarized here in particular, it is likely that many studies employing single acoustic measures of convergence will result in null findings that will fail to be reported in the literature. Therefore, there are practical reasons to include perceptual assessment of phonetic convergence in addition to the enhanced reliability and validity that this approach affords. Going forward, studies of phonetic convergence should adopt both perceptual and acoustic measures together.

## Future directions

A more comprehensive paradigm for exploring phonetic convergence integrates acoustic and perceptual measures in the same study. Previously, measures of articulation rates and/or vowel formants have been found to differ from perceived phonetic convergence during conversational interaction or among college roommates (Pardo, 2010; Pardo et al., 2010, 2012, 2013a). However, those studies either used ordinary linear regression or correlation analyses, along with traditional analyses of variance. A recent study of phonetic convergence in shadowed speech assessed the relative contribution of distinct acoustic-phonetic parameters to perceived phonetic convergence (Pardo et al., 2013b). This approach demonstrates the utility of comparing multiple acoustic measures to perceived phonetic convergence using mixed-effects regression modeling (Baayen, 2008; Baayen et al., 2008; Jaeger, 2008; Barr et al., 2013).

In this study, phonetic convergence during a shadowing task was measured in 4 ways, using a perceptual AXB task and 3 acoustic measures. A total of 20 talkers provided baseline and shadowed versions of 80 monosyllabic words produced by 20 model talkers in same-sex pairings. The words were used in an AXB perceptual test of phonetic convergence. Three acoustic measures representing some of the most commonly reported attributes in the literature were compared to the perceptual assessment of phonetic convergence: Duration, F0, and Vowel Formants. These data demonstrate the variable nature of convergence across multiple dimensions. While the perceptual AXB data yielded results that were consistently significant at all scales of analysis, the acoustic measures of convergence were highly variable across words and shadowers, yielding inconsistent results. In particular, none of the acoustic parameters demonstrated significant convergence on average. Moreover, the levels of convergence in Duration, F0 and Vowel Formants were markedly different from each other and from perceived convergence across talkers. If any one of the acoustic measures had been the sole focus of the study, then the null results would not have contributed interpretable findings. However, the AXB perceptual data can be used to calibrate the relative contribution of each acoustic measure to phonetic convergence by using them as predictors in a mixed-effects regression model of the perceptual data.

Mixed-effects modeling can accommodate this variability because it treats all of the data on an item-by-item basis—all sources of variability are entered into the model without collapsing. By modeling listeners, items, and talkers as random sources simultaneously, mixed-effects regression harnesses their variability to derive measures of the relative contribution of each fixed effect in the model. Therefore, these models can assess the relative contributions of individual acoustic-phonetic attributes to perceived phonetic convergence, as well as other manipulated variables.

When modeling acoustic measures as predictors of the perceptual data, the best-fitting model depended on including all three acoustic measures together. According to the parameter weights associated with the model, perceived phonetic convergence was most strongly associated with variability in Duration, followed by F0 and Vowel Formants. Furthermore, the combined effects of all three parameters is necessary to explain phonetic convergence, as models that did not include one of the measures did not maximize fit. However, the resulting model leaves much to be explained, and a more comprehensive set of acoustic measures would likely improve the fit of the model, and ultimately an understanding of the set of acoustic-phonetic attributes that are most often and most strongly employed in phonetic convergence. Future investigations would benefit from taking a more comprehensive approach that assesses the relative contributions of multiple dimensions to phonetic convergence in speech production.

Perceptual measures of phonetic convergence provide a holistic assessment of change in multiple acoustic-phonetic parameters simultaneously. A talker who converges to (or diverges from) a model/partner does so on multiple attributes within a single item, and the relative convergence of any single attribute varies across words. There is no one acoustic attribute that serves as a metric of convergence across words and talkers. Therefore, perceptual assessment is crucial to any investigation of phonetic convergence because listeners effectively collapse across the multidimensional space when making similarity judgments (Pardo and Remez, 2006).

Studies of phonetic convergence that only focus on a single acoustic-phonetic attribute provide only a glimpse of the phenomenon. Because of the inherently variability in speech production and perception, such glimpses are likely to yield an incomplete (at best) or inaccurate (at worst) characterization of phonetic convergence. Mixed-effects modeling of perception can be used to calibrate the relative prominence of multiple dimensions in speech production. The current paper demonstrates the utility of incorporating multiple acoustic measures in concert with perceptual assessment of phonetic convergence.

### Conflict of interest statement

The author declares that the research was conducted in the absence of any commercial or financial relationships that could be construed as a potential conflict of interest.
